# Comparative Study on Nanotoxicity in Human Primary and Cancer Cells

**DOI:** 10.3390/nano12060993

**Published:** 2022-03-17

**Authors:** In Young Kim, Minjeong Kwak, Jaeseok Kim, Tae Geol Lee, Min Beom Heo

**Affiliations:** Nano-Safety Team, Safety Measurement Institute, Korea Research Institute of Standards and Science (KRISS), Yuseong-gu, Daejeon 34113, Korea; inyoungkim@kriss.re.kr (I.Y.K.); kwakmj@kriss.re.kr (M.K.); jaeseok.kim@kriss.re.kr (J.K.); tglee@kriss.re.kr (T.G.L.)

**Keywords:** nanotoxicity, nanomaterial, cancer cells, silica nanoparticles

## Abstract

Nanomaterial toxicity tests using normal and cancer cells may yield markedly different results. Here, nanomaterial toxicity between cancer and primary human cells was compared to determine the basic cell line selection criteria for nanomaterial toxicity analyses. Specifically, we exposed two cancer (A549 and HepG2) and two normal cell lines (NHBE and HH) cell lines to SiO_2_ nanoparticles (NPs) and evaluated the cytotoxicity (MTS assay), cell death mode, and intracellular NP retention. MTS assay results revealed higher sensitivity of HH cells to SiO_2_ NPs than HepG2 cells, while no difference was observed between NHBE and A549 cells. In addition, SiO_2_ NPs primarily induced necrosis in all the cell lines. Moreover, we evaluated NP accumulation by treating the cell lines with fluorescein-isothiocyanate-labeled SiO_2_ NPs. After 48 h of treatment, less than 10% of A549 and HepG2 cells and more than 30% of NHBE and HH cells contained the labeled NPs. Collectively, our results suggest that cell viability, death mode, and intracellular compound accumulation could be assessed using cancer cells. However, the outcomes of certain investigations, such as intracellular NP retention, may differ between cancer and normal cells.

## 1. Introduction

Nanotechnology has become a valuable and effective essential technology across several fields in recent years. Various nanomaterials, with sizes smaller than those of human cells, are widely used in cosmetics [1,2], sunscreens [3], food packaging [4], and pharmaceuticals [5]. However, their small size can make them toxic, posing risks to human health and safety [6,7,8].

As one of the most-produced nanoparticles (NPs) globally, silica NPs (SiNPs) are used in all aspects of life, including agriculture, food, and consumer goods [9]. In addition, the biocompatibility and stability of SiNPs make them promising candidates in various biomedical fields, such as gene carriers, drug delivery, and molecular imaging [10,11]. However, previous studies have reported on the toxic effects of SiNPs on essential organs such as the liver, lungs, brain, and kidneys [12,13]. Thus, extensive use has increased the risk of SiNPs exposure in humans.

Therefore, it is essential to investigate the safety of nanomaterials. Traditionally, toxicity studies using animals have been conducted to estimate the adverse effects of chemicals and nanomaterials on humans. However, many countries, particularly those in Europe, have recently begun regulating and limiting animal studies for ethical reasons; therefore, other test methods that estimate chemical or nanomaterial toxicity reliably are required [14]. Accordingly, human cell models that reduce unnecessary animal sacrifice and lower costs have been established. Presently, nanomaterial toxicity is being investigated using human cancer cells or immortalized cells [15,16]. Owing to their low cost, ease of handling, and excellent reproducibility, human cancer cells have considerable advantages in the rapid toxicity screening of nanomaterials or chemicals over other cells. However, unlike normal cells, cancer cells have unique characteristics, such as proliferating indefinitely due to excessive cell division from lack of cell cycle control and the apoptosis function [17]. In addition, cancer cells acquire energy by metabolizing glucose. The glycoprotein structure expressed on their membrane surface significantly differs from those on normal cells [18,19], resulting in studies raising concerns regarding the misjudgment of toxicity outcomes. Studies have associated nanomaterial toxicity with the cell lines used [20,21]. Therefore, results can be entirely different from those of normal cells when analyzing cell viability and the mechanism underlying the toxicity of nanomaterials using cancer cells [22].

Here, we compared the differences in nanomaterial toxicity between cancer and primary human cells. The liver and lungs, most affected by toxic substances, were the target organs in the current study. We used A549 (human lung cancer cell line) and NHBE cells (normal human bronchial epithelial cell line) as the lung model cells, and HepG2 (human liver cancer cell line) and HH cells (normal human hepatocyte line) as the liver model cells. Our goal was to aid the selection of cell lines for nanomaterial toxicity studies by comparing the toxicity test results and confirming differences in modes of cell death caused by SiO_2_ NPs using different target organ cell lines.

## 2. Materials and Methods

### 2.1. Materials and Reagents

For the experiments, we used 20 nm SiO_2_ NPs, which are certified reference materials (CRM, 301-01-002) produced by the Korea Research Institute of Standards and Science (Daejeon, Korea). Cadmium sulfate (CdSO_4_, #383082) and stauroporine (STS, #S5921) were purchased from Sigma-Aldrich (St. Louis, MO, USA).

### 2.2. Preparation and Characterization of SiO_2_ NPs

The SiO_2_ NPs were dispersed in deionized water or the Dulbecco’s modified Eagle medium (DMEM; #LM001-05, Welgene, Gyeongsan, South Korea) via vortexing for 1 min. The morphology and size of the primary SiO_2_ NPs were analyzed using transmission electron microscopy (TEM; JEM-ARM200F, JEOL Ltd., Tokyo, Japan) and scanning electron microscopy (SEM; Gemini SEM 500, Carl Zeiss, Oberkochen, Germany). The size distribution of the SiO_2_ NPs was measured using dynamic light scattering (DLS; Nano ZS90, Malvern Panalytical, Worcestershire, UK) and scanning mobility particle sizer (SMPS; TSI Incorporated, Shoreview, MN, USA). The zeta-potential was measured using the Zetasizer instrument (Nano ZS90, Malvern, UK).

### 2.3. Cell Culture

A549 and HepG2 cells, purchased from the American Type Culture Collection (Manassas, VA, USA), were cultured in DMEM supplemented with 10% fetal bovine serum (FBS; #SH30084.03, HyClone™, Marlborough, MA, USA) and 1% penicillin-streptomycin (#LS202-02, Welgene™). NHBE cells were purchased from Lonza™ (Alps, Switzerland). These cells were cultured in bronchial epithelial cell growth basal medium (#CC-3171, Lonza™) containing a supplement pack (#CC-4175, Lonza™). HH cells purchased from ScienCell™ (Carlsbad, CA, USA) were cultured in hepatocyte medium (#5201, ScienCell™) containing 5% FBS (#0025, ScienCell™), 1% penicillin-streptomycin (#0503, ScienCell™), and hepatocyte growth supplement (#5252, ScienCell™). All cells were incubated at 37 °C in a humidified atmosphere containing 5% CO_2_. All cells were expanded after passage number three and then frozen in liquid nitrogen. After thawing, cells having passage numbers between 4 and 7 were used for the toxicity test.

### 2.4. Cytotoxicity Assay and Statistical Analysis

All cells were seeded at a density of 1.0 × 10^4^ cells/well onto a 96-well plate (#3596, Corning^®^, Glendale, AZ, USA) and incubated for 24 h. After washing the cells with phosphate-buffered saline (PBS; LB001-02, Welgene™), they were treated with the prepared SiO_2_ NPs, dispersed in serum-free DMEM, for 4 h. Cytotoxicity was quantified using MTS assay (CellTiter96^®^ AQueous One Solution Cell Proliferation Assay kit, Promega, Madison, WI, USA) according to the manufacturer’s instructions. The 96-well plates were then incubated at 37 °C in a humidified atmosphere containing 5% CO_2_ for 1 h. Cell viability was calculated using Equation (1). IC_50_ values of the NPs were calculated using the SoftMax Pro software (Molecular Devices, San Jose, CA, USA).

(1) $$\mathrm{Cell}\;\mathrm{viability}\;\left(\%\right)\;=\frac{\mathrm{Absorbance}\;\mathrm{value}\;\mathrm{of}\;\mathrm{test}\;\mathrm{cells}}{\mathrm{Absorvance}\;\mathrm{value}\;\mathrm{of}\;\mathrm{control}\;\mathrm{cells}}\times 100$$

The results of all experiments were statistically analyzed using GraphPad Prism software (version 7.0; GraphPad Software Inc., San Diego, CA, USA) and represented as the mean and standard error of independent experiments (*n* = 12). The normality of the data was assessed using the Kolmogorov–Smirnov test, and equal variance using Bartlett’s test. For normally distributed data, statistical differences were determined using variance analysis followed by Bonferroni’s multiple comparison test; else, the Kruskal–Wallis test was performed, followed by Dunn’s test.

### 2.5. Investigation of Cell Death Mode

An Annexin V–Fluorescein Isothiocyanate (FITC) Apoptosis Detection kit (#130-092-052, Miltenyi Biotec, Bergisch Gladbach, Germany) was used to determine apoptosis according to the manufacturer’s instructions. Using this kit, we washed the cells twice with 1 × binding buffer and then resuspended in 100 µL of 1 × binding buffer per 1.0 × 10^6^ cells. Next, the cells were incubated with 10 μL of Annexin V-FITC in the dark at room temperature for 15 min and washed with 1 mL of 1 × binding buffer. Thereafter, the cells were stained with propidium iodide (PI), before resuspending in 500 µL of 1 × binding buffer per 1.0 × 10^6^ cells. We added 5 μL of PI solution prior to analysis by fluorescence microscopy or flow cytometry. Fluorescence microscopy was performed using a confocal laser scanning microscope (Olympus, Tokyo, Japan), and flow cytometry was performed using Eclipse™ (Sony Biotechnology, San Jose, CA, USA).

### 2.6. Visualization of FITC-Labeled 20 nm SiO_2_ NPs Using Fluorescence Microscopy

Cells (5.0 × 10^4^ cells/well), seeded on a coverslip and incubated for 48 h, were treated with FITC-labeled 20 nm SiO_2_ NPs for 4 h and fixed with 4% paraformaldehyde for 20 min. Next, the cells were washed twice with PBS and observed using fluorescence microscopy (Leica Microsystems, Wetzlar, Germany).

### 2.7. FITC-Labeled 20 nm SiO_2_ NPs Uptake Analysis Using Flow Cytometry

For uptake analysis, cells (2.5 × 10^5^ cells/well) were first seeded on 6 well plates and incubated. The next day, FITC-labeled 20 nm SiO_2_ NPs were co-cultured with the cells for 4 h. The cells were washed twice with PBS and analyzed using flow cytometry. The live single-cell population was gated in a plot of FSC versus SSC after excluding cell FITC-negative cell population was gated in a plot of FSC versus FITC in the untreated sample. In all samples, the percentage of FITC-positive cells in a single-cell population decided the intracellular localization of FITC-SiO_2_ NPs.

## 3. Results

### 3.1. Characterization of SiO_2_ NPs

**Figure 1:**
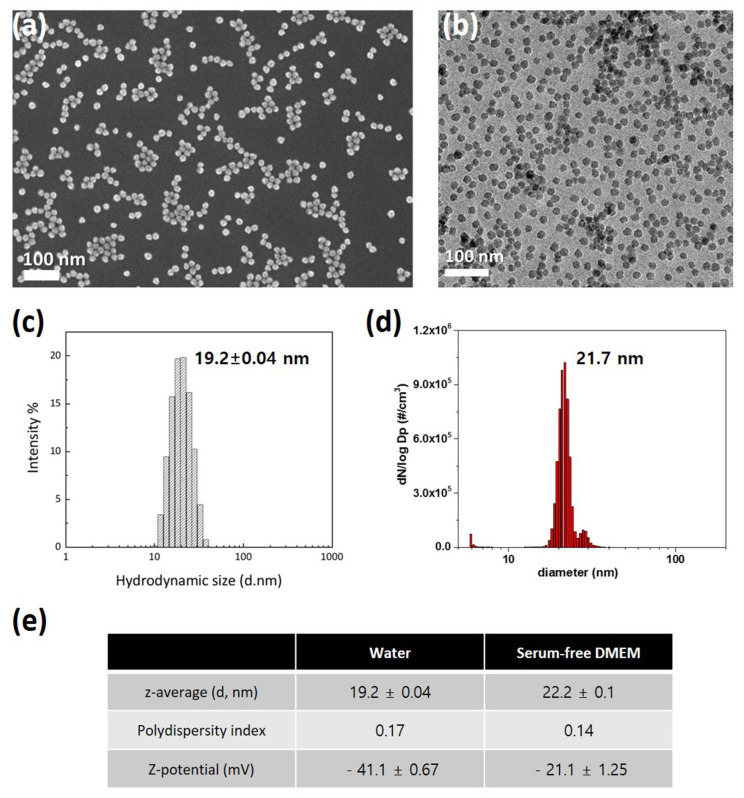
Characterization of 20 nm SiO_2_ NPs: (**a**) Scanning electron microscopy image; (**b**) Transmission electron microscopy image; (**c**) Dynamic light scattering size analysis; (**d**) Scanning mobility particle sizer analysis; (**e**) Performance comparison in deionized water and serum-free DMEM.

For characterization of SiO_2_ NPs in deionized water, their morphology and size were investigated using electron microscopy. SiO_2_ NPs exhibit uniform spherical morphology and size distribution in SEM (Figure 1a) and TEM (Figure 1b) images. DLS (Figure 1c) and SMPS (Figure 1d) determined the hydrodynamic sizes of SiO_2_ NPs, which were 19.2 and 21.7 nm, respectively. Since NPs can have significant variations in size distribution in biological environments, we compared SiO_2_ NPs in deionized water and serum-free DMEM. Unexpectedly, z-average and polydispersity index are similar in the two dispersions (Figure 1e). In addition, the zeta-potential show negative charges in both conditions (Figure 1e). These results indicate that SiO_2_ NPs are monodispersed without aggregation.

### 3.2. Cytotoxicity of SiO_2_ NPs

**Figure 2:**
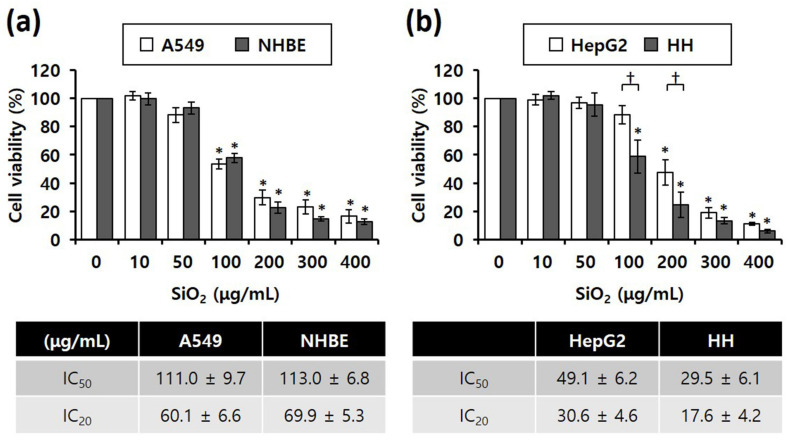
MTS cytotoxicity assay results showing the different inhibitory concentrations of 20 nm SiO_2_ NPs against cells subjected to NP treatment for 4 h (total number of replicates = 12); (**a**) Lung-derived cells. * *p* < 0.005, compared with untreated cells. (**b**) Liver-derived cells. * *p* < 0.005, compared with untreated cells; † *p* < 0.05, compared with HepG2 cells.

Our study compared SiO2 NPs toxicity between cancer (A549 and HepG2) and normal cell lines (NHBE and HH), MTS assay results revealed that the viability of all four cell lines significantly decreased depending on the concentration of SiO_2_ NPs in the cells (Figure 2). Notably, IC_50_ values of SiO_2_ NPs were almost similar between the lung-derived A549 and NHBE cells (Figure 2a), suggesting that there was no difference in toxicity caused by SiO_2_ NPs between the two cell lines. However, in the liver-derived cell lines, the IC_50_ values of SiO_2_ NPs in normal HH cells were approximately 1.5-fold higher than that in HepG2 cells, suggesting that the higher sensitivity of HH cells to SiO_2_ NPs was higher than that of HepG2 cells (Figure 2b).

### 3.3. Cell Death Mode

**Figure 3:**
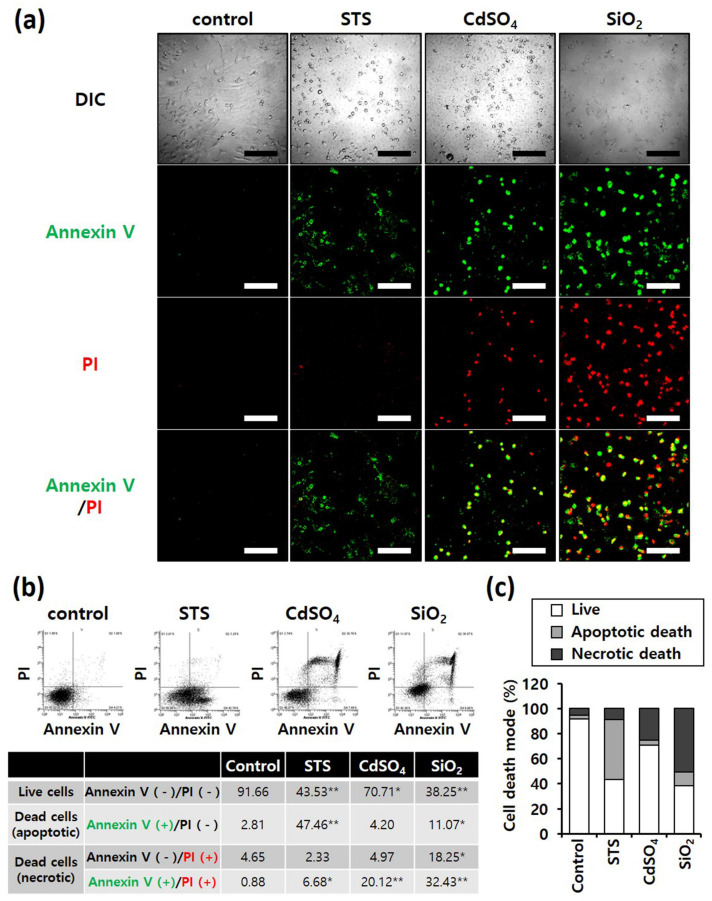

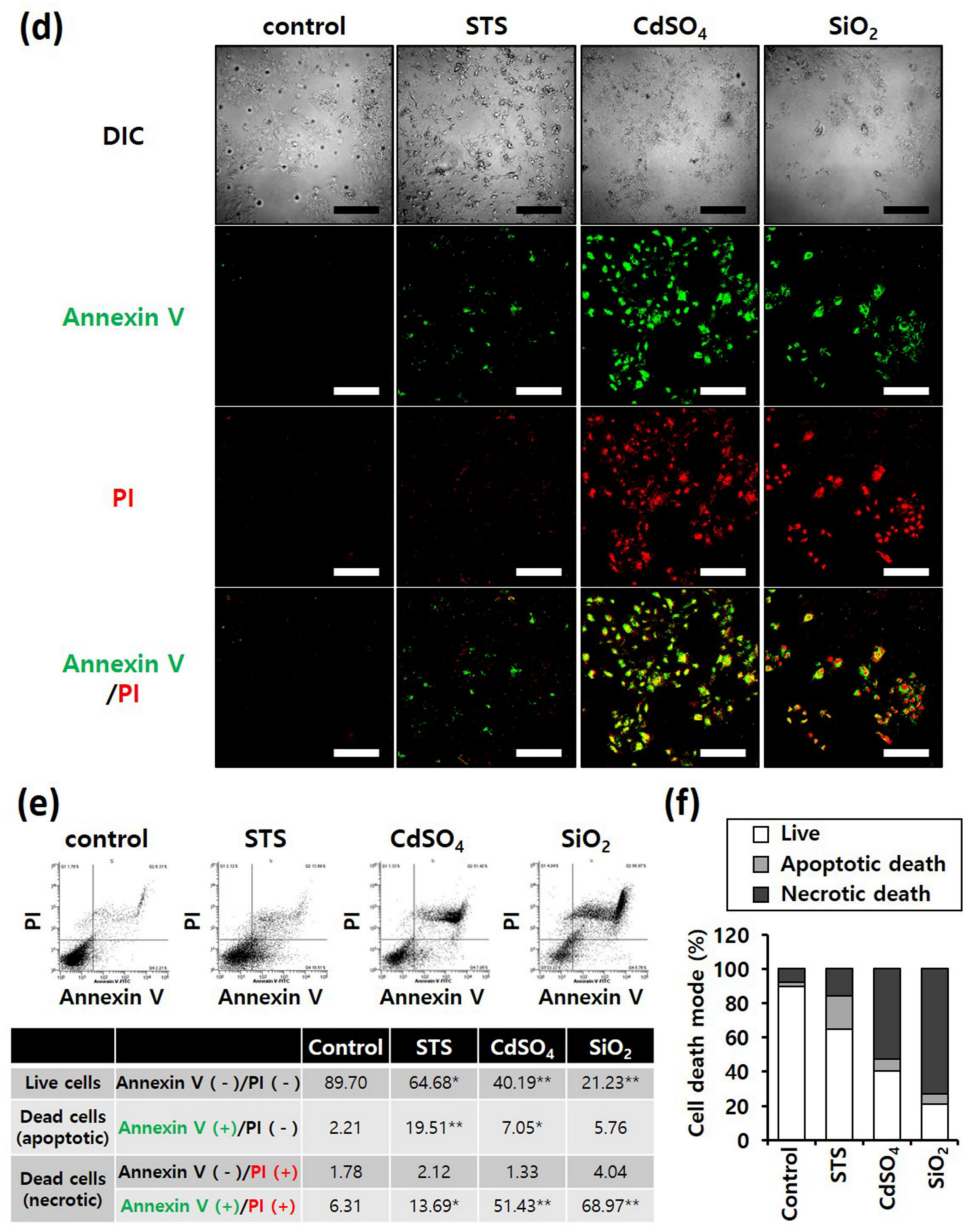
Annexin-V/PI double-staining assay of A549 and NHBE cells; After treating A549 and NHBE cells with SiO_2_ NPs (at IC50) and two positive controls, the cells were stained with annexin V-fluorescein isothiocyanate and propidium iodide and analyzed by fluorescence microscopy and flow cytometry. CdSO_4_ (1.0 mM) was used to induce necrosis, and staurosporine (STS; 1.0 μM) was used to induce apoptosis; replicate number = 3. (**a**,**d**) Confocal fluorescence microscopy images; scale bars represent 200 μm. (**b**,**e**) Flow cytometry analysis. * *p* < 0.05, compared with control; ** *p* < 0.005, compared with control. (**c**,**f**) Percentage distribution of necrotic, apoptotic, and viable cells.

**Figure 4:**
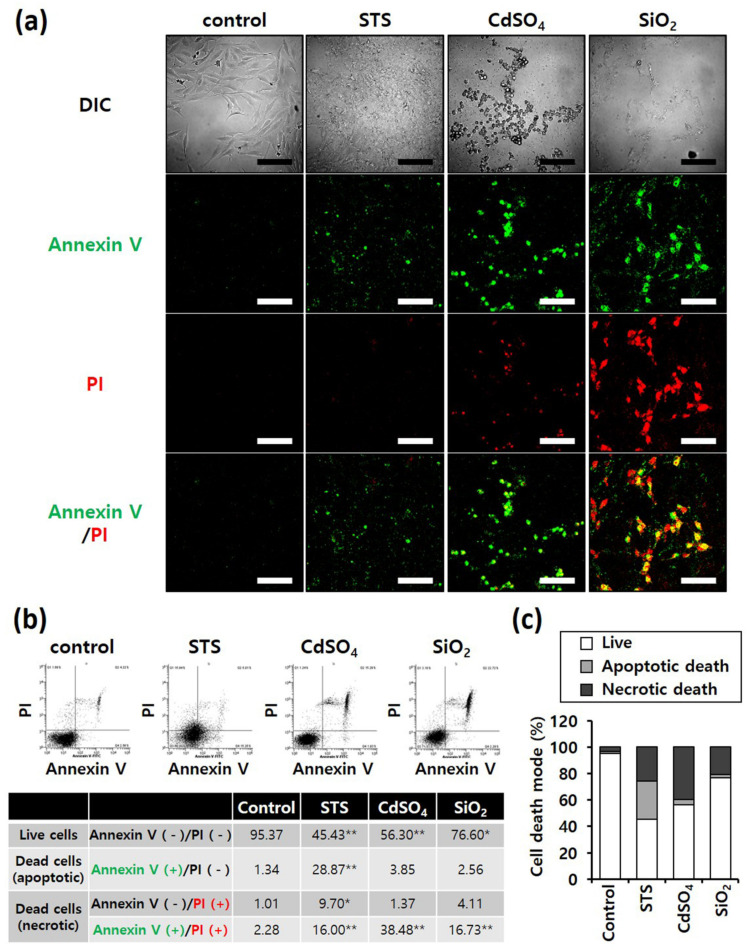

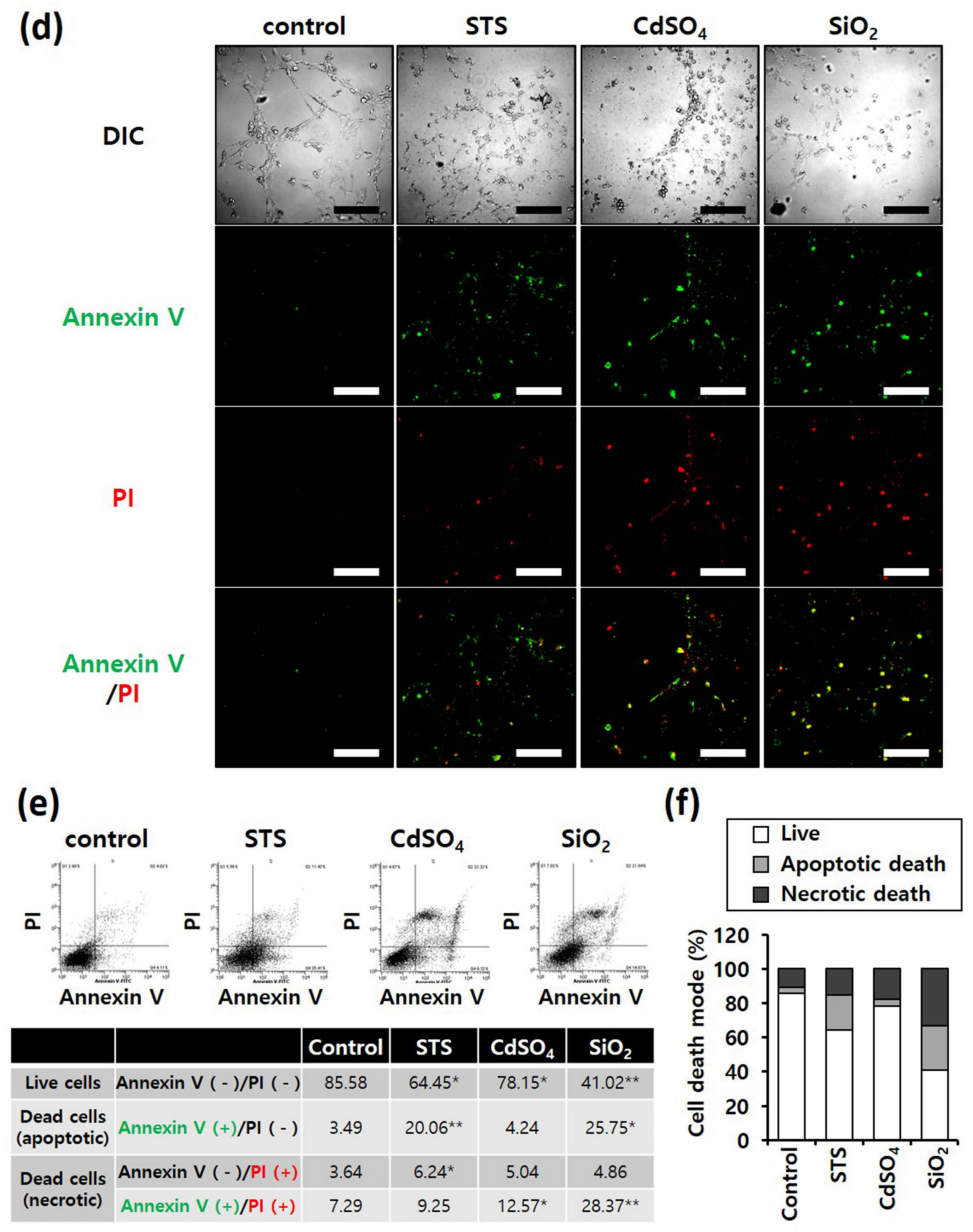
Annexin-V/PI double-staining assay of HepG2 and HH cells; After treating HepG2 and HH cells with SiO_2_ NPs (at IC50) and two positive controls, the cells were stained with annexin V-fluorescein isothiocyanate and propidium iodide and analyzed by fluorescence microscopy and flow cytometry. CdSO_4_ (1.0 mM) was used to induce necrosis, and staurosporine (STS; 1.0 μM) was used to induce apoptosis; replicate number = 3. (**a**,**d**) Confocal fluorescence microscopy images; scale bars represent 200 μm. (**b**,**e**) Flow cytometry analysis. * *p* < 0.05, compared with control; ** *p* < 0.005, compared with control. (**c**,**f**) Percentage distribution of necrotic, apoptotic, and viable cells.

Annexin V/PI staining was performed to determine differences in the mode of death caused by SiO_2_ NPs between cancer and normal cells. STS was used as a positive control for apoptosis, and CdSO_4_ for necrosis. In distinguishing the mode of cell death, Annexin V(+)/PI(−) cell populations were considered apoptotic, while Annexin V(−)/PI(+) or Annexin V(+)/PI(+) cell populations were considered necrotic. Fluorescence microscopy shows that the STS treatment increased green fluorescence intensity, while CdSO_4_ treatment increased green and red fluorescence intensities in the lung-derived cell lines, A549 and NHBE (Figure 3a,d). Green and red fluorescence intensities were also significantly increased by SiO_2_ NP treatment. Next, the mode of cell death was analyzed by sorting cells according to fluorescence using flow cytometry. STS primarily induced apoptosis, while CdSO_4_ and SiO_2_ NPs induced necrosis in A549 cells (Figure 3b,c). In NHBE cells, STS induced a mixture of apoptosis and necrosis, while CdSO_4_ primarily induced necrosis. SiO_2_ NPs induced necrosis in NHBE cells (Figure 3e,f). These results suggest that the mode of death induced by SiO_2_ NPs was similar in A549 and NHBE cells. Similar to the lung-derived cell lines, STS treatment increased green fluorescence intensity, while CdSO_4_ and SiO_2_ NPs increased green/red fluorescence intensity in HepG2 and HH cell lines (Figure 4a,d). Flow cytometry analysis revealed that STS induced both apoptosis and necrosis, while CdSO_4_ mostly induced necrosis (Figure 4b,c,e,f). In contrast, SiO_2_ NP treatment mostly induced necrosis in HepG2 cells and apoptosis and necrosis in HH cells (Figure 4b,c,e,f). These results suggest that the mode of death induced by SiO_2_ NPs in HepG2 and HH cells differ partially.

### 3.4. Examination of SiO_2_ NPs Retention in Cells

**Figure 5:**
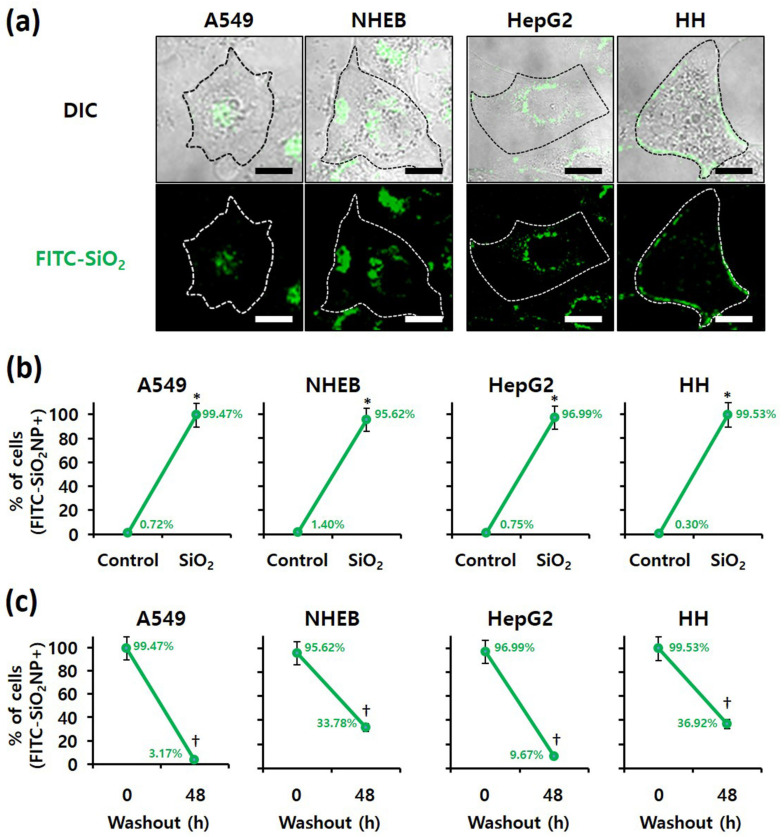
Examination of SiO_2_ NPs retention in cells; Localization of fluorescein isothiocyanate (FITC)-labeled 20 nm SiO_2_ NPs in each cell line, as determined by (**a**) confocal fluorescence microscopy (scale bars represent 10 µm) and (**b**) flow cytometry after 4 h of treatment. * *p* < 0.005, compared with control. (**c**) Retention of FITC-labeled 20 nm SiO_2_ NPs in each cell line, as determined by flow cytometry at 0 and 48 h after 4 h of treatment. † *p* < 0.005, compared with washout for 0 h.

Because intracellular nanomaterials can majorly cause cytotoxicity, the intracellular accumulation of SiO_2_ NPs was confirmed. First, confocal microscopy revealed FITC-labeled SiO_2_ NPs in the four cell lines when treated with these NPs (Figure 5a). In addition, the proportion of cells containing FITC-labeled SiO_2_ NPs, determined by flow cytometry, revealed that more than 95% of all A549, NHBE, HepG2, and HH cells contained FITC-labeled SiO_2_ NPs (Figure 5b). We conclude that SiO_2_ NPs were internalized in the four cell lines without difficulty. For analysis of residual NPs, the culture medium was changed after 4 h of FITC-labeled SiO_2_ NP treatment (Figure 5c). The number of cells containing FITC-labeled SiO_2_ NPs was counted using flow cytometry at 0 and 48 h after treatment. After 48 h of treatment, FITC-labeled SiO_2_ NPs were less than 10% in A549 and HepG2 cancer cells and more than 30% in NHBE and HH normal cells. Thus, the removal rate of NPs from cancer cells was markedly faster than that from normal cells. Therefore, regardless of their origin, cancer and normal cells differ.

## 4. Discussion

When screening NPs, selecting the appropriate cell type is critical. Primary cells have several disadvantages, such as limited lifespan, specific culture conditions, and batch-to-batch differences [23,24]. It has been shown that NP-induced effects can vary according to species, tissue, and cell line in many groups. Therefore, cancer cell lines are used as a universal model in nanosafety evaluation. However, uncertainty can be introduced depending on the cell line type and characteristics when generalizing data [25,26,27,28]. A few studies have compared normal and cancer cells in the same species and tissue. Ekstrand-Hammarstrom and colleagues compared the cellular uptake and response of TiO_2_ NPs to NHBE (primary), BEAS-2B (human bronchial epithelial cell line, immortalized), and A549 (cancerous). NHBE, BEAS-2B, and A549 cells had similar cell viability and oxidative stress induction, but different proinflammatory responses [29]. Kermanizadeh and co-workers showed similar cellular responses to ZnO, MWCNT, Ag, and TiO_2_ NPs in primary human hepatocytes (normal) and C3A cell lines (clonal derivative of HepG2, hepatocellular carcinoma) [30].

This study is the first to compare the cytotoxic effects and cellular localization of SiO_2_ NPs in cancer cells (lung-A549, liver-HepG2) and corresponding primary human cells (lung-NHBE, liver-HH). While lung-origin cells had similar sensitivity to SiO_2_ NPs (Figure 2 and Figure 3), hepatic-origin had higher HH sensitivity to SiO_2_ NPs than HepG2 (Figure 2 and Figure 4). In addition, we confirmed that the degree of intracellular accumulation of SiO_2_ NPs was similar in the four cell types (Figure 5). Therefore, we infer no difficulty using cancer cells for short-term cytotoxicity screening of NPs, while probably abandoning the notion of representing all cells.

We showed that the clearance of intracellular SiO_2_ NPs was much faster in cancer cells (Figure 5c). The pathway of eliminating intracellular NPs compared to the cellular uptake mechanism is less well known. Intracellular NP concentration can be reduced through (1) apoptosis, (2) cell proliferation, (3) NP diffusion, (4) lysosomal degradation, and (5) exocytosis [31]. Since intracellular NPs fail to reach sufficient concentrations to cause cell death, cell death or proliferation need not be the main mechanism for cellular excretion of NPs [31]. Because cancer cells have a significantly higher proliferation rate than normal cells, the intracellular particle concentration is reduced more efficiently in our study. In addition, the difference in the ability of lysosomes between normal and cancer cells may affect the SiO_2_ NP removal rate. Lysosomes remove intracellular NPs through direct degradation [32,33] and exocytosis [34,35,36,37,38,39]. Many researchers argue that lysosomal stability is necessary to clear intracellular NP [34,35,36,40]. During transformation into malignancy, cancer cells have enhanced lysosomal functions, such as biosynthesis, hydrolase activity, and exocytosis, to obtain substances necessary for assimilation and catabolism [41]. Therefore, we suggest that cancer cells with improved lysosomal function can reduce the intracellular particle concentration more efficiently than normal cells. In addition, cell viability and response between normal and cancer cells may differ upon long-term exposure to low SiO_2_ NP concentration because the accumulated NPs can destroy organelles such as lysosomes and reach the cytoplasm.

Many studies on the toxic effects of SiNPs have been performed using in vivo models. However, awareness of interspecies differences [42,43,44] and restrictions on animal ethics [EU-Directive 2010/63] drive interest in developing human-based in vitro models. Despite their drawbacks in representing effects on humans or tissues due to their simplicity, they can be a valuable source in evaluating toxicity and mechanical data for drugs, chemicals, and NPs. Therefore, finding a cell line that is more similar to the tissue of origin is important in developing a standardized protocol for nanosafety evaluation. Although the current study cannot represent all cells, it provides a rationale for using cancer cells for short-term nanosafety screening. More research is needed for more accurate results on clinical trials, which requires data accumulation.

## 5. Conclusions

This study determines the possibility of using cancer cells instead of normal cells to evaluate the safety of nanomaterials in vitro. Viability, mode of death, and intracellular compound accumulation were compared between cancer and normal cells after treating them with SiO_2_ NPs. The results suggested that cancer cells can be used to investigate the above parameters, having short evaluation periods. However, long-term evaluations, such as recovery after exposure to NPs, may differ between cancer and normal cells. In conclusion, the standardization of nanomaterial safety evaluation is required in future studies because the effect of NPs may vary depending on the origin of the test cells.

## Data Availability

The data presented in this study are available on request from the corresponding author.
